# Sediment Bacterial Community Structure under the Influence of Different Domestic Sewage Types

**DOI:** 10.4014/jmb.2004.04023

**Published:** 2020-06-24

**Authors:** Lei Zhang, Mengli Xu, Xingchen Li, Wenxuan Lu, Jing Li

**Affiliations:** 1 School of Civil Engineering and Architecture, Chuzhou University, Chuzhou 239000, P.R. China; 2 Fisheries Research Institute, Anhui Academy of Sciences, Hefei 230001, P.R. China

**Keywords:** Urban river, domestic sewage, bacterial community, metabolic function, co-occurrence network

## Abstract

Sediment bacterial communities are critical to the biogeochemical cycle in river ecosystems, but our understanding of the relationship between sediment bacterial communities and their specific input streams in rivers remains insufficient. In this study, we analyzed the sediment bacterial community structure in a local river receiving discharge of urban domestic sewage by applying Illumina MiSeq high-throughput sequencing. The results showed that the bacterial communities of sediments samples of different pollution types had similar dominant phyla, mainly Proteobacteria, Actinobacteria, Chloroflexi and Firmicutes, but their relative abundances were different. Moreover, there were great differences at the genus level. For example, the genus *Bacillus* showed statistically significant differences in the hotel site. The clustering of bacterial communities at various sites and the dominant families (*i.e*., *Nocardioidaceae*, and *Sphingomonadaceae*) observed in the residential quarter differed from other sites. This result suggested that environmentally induced species sorting greatly influenced the sediment bacterial community composition. The bacterial co- occurrence patterns showed that the river bacteria had a nonrandom modular structure. Microbial taxonomy from the same module had strong ecological links (such as the nitrogenium cycle and degradation of organic pollutants). Additionally, PICRUSt metabolic inference analysis showed the most important function of river bacterial communities under the influence of different types of domestic sewage was metabolism (*e.g*., genes related to xenobiotic degradation predominated in residential quarter samples). In general, our results emphasize that the adaptive changes and interactions in the bacterial community structure of river sediment represent responses to different exogenous pollution sources.

## Introduction

Rivers are the major source of renewable water for freshwater ecosystems, and they have important ecological and economic value [1-3]. Currently, with the rapid growth of urbanization and population, much domestic sewage is discharged into rivers [4, 5]. Untreated or only partially treated domestic sewage contains a large number of pollutants, including antibiotics [6, 7], pathogenic microorganisms [4, 8], organic pollutants [9], suspended solids [10] and nutrients [11]. The chemical degradation of these pollutants may cause the eutrophication of the river, decrease biological diversity, and accelerate the spread of pathogens in natural water systems [5, 12, 13], thereby exposing water users to serious health risks. A more serious issue is the slow accumulation of pollutants in the water sediment. Once released, the pollutants can be suspended in the water, which will again affect the quality of the water column and increase the risk of human infection [14]. Therefore, a sensitive and robust indicator is needed to identify and monitor changes within river sediments under the influence of domestic sewage.

Fortunately, sediment microbial properties, especially bacterial diversity and community structure, represent very promising candidate bioindicators of polluted rivers [15]. Sedimentary bacteria are highly responsive to environmental changes [16], and they are the first to interact with dissolved matter [17]. Domestic sewage containing organic and inorganic pollutants significantly changes the basic parameters of river sediment, such as the nitrogen [18], phosphorus [19], and dissolved oxygen [4] content, thus further reshaping the bacterial community structure.

There has long been keen interest to investigate not only the physical and chemical properties of rivers (such as heavy metals [20], hydrocarbons [21] or nutrients [22] ), but also the composition of microbial communities in extreme or complex river ecosystems, their structure, and the relationship between diversity and the environment [23-26]. However, the contribution of these complex pollution sources to river ecosystems is comprehensive [1, 62]. Therefore, exploring a single source of pollution can provide an accurate understanding of the relationship between bacterial communities in rivers and their specific input streams. Studies have shown that domestic wastewater is a critical issue in public health and disease prevention worldwide [28]. In recent years, research examining the impact of domestic pollution on river biodiversity has mainly focused on the impact on aquatic plants [29, 30] or plankton [31] and has poorly addressed bacterial communities in river sediments.

Abiotic environmental factors affect microbial community function and the ecological mechanisms of interspecific interactions, excluding the composition and structure of microbial communities [32]. Research has shown that the function of the microbial community can provide a new perspective for the study of ecosystem function changes under different environmental conditions, including those related to the biogeochemical cycles of C, N, and P, as well as biomass production [33-35]. In addition, it has been proposed that microbial communities usually have nonrandom co-occurrence patterns and a modular structure [36], which strongly suggests that biological interactions play an important role in controlling community aggregation and ecosystem functions.

We studied the composition of bacterial communities in river sediment and predicted the potential functional consequences under the influence of different domestic sewage types by uniting the bacteria-environment relationship and bacterial-bacterial interactions. In this study, we used the Qingliu River and its four typical domestic pollution input streams as an example. Our specific goals were (1) to explore the reasons for the difference of bacterial diversity composition in river sediments affected by different domestic sewage and (2) to investigate the correlation between the bacterial diversity of river sediments and their habitats under different domestic sewage pollution. The results from this study can provide a reference for the rational management and conservation of river ecosystems.

## Materials and Methods

### Description of the Study Sites and Sample Collection

**Fig. 1:**
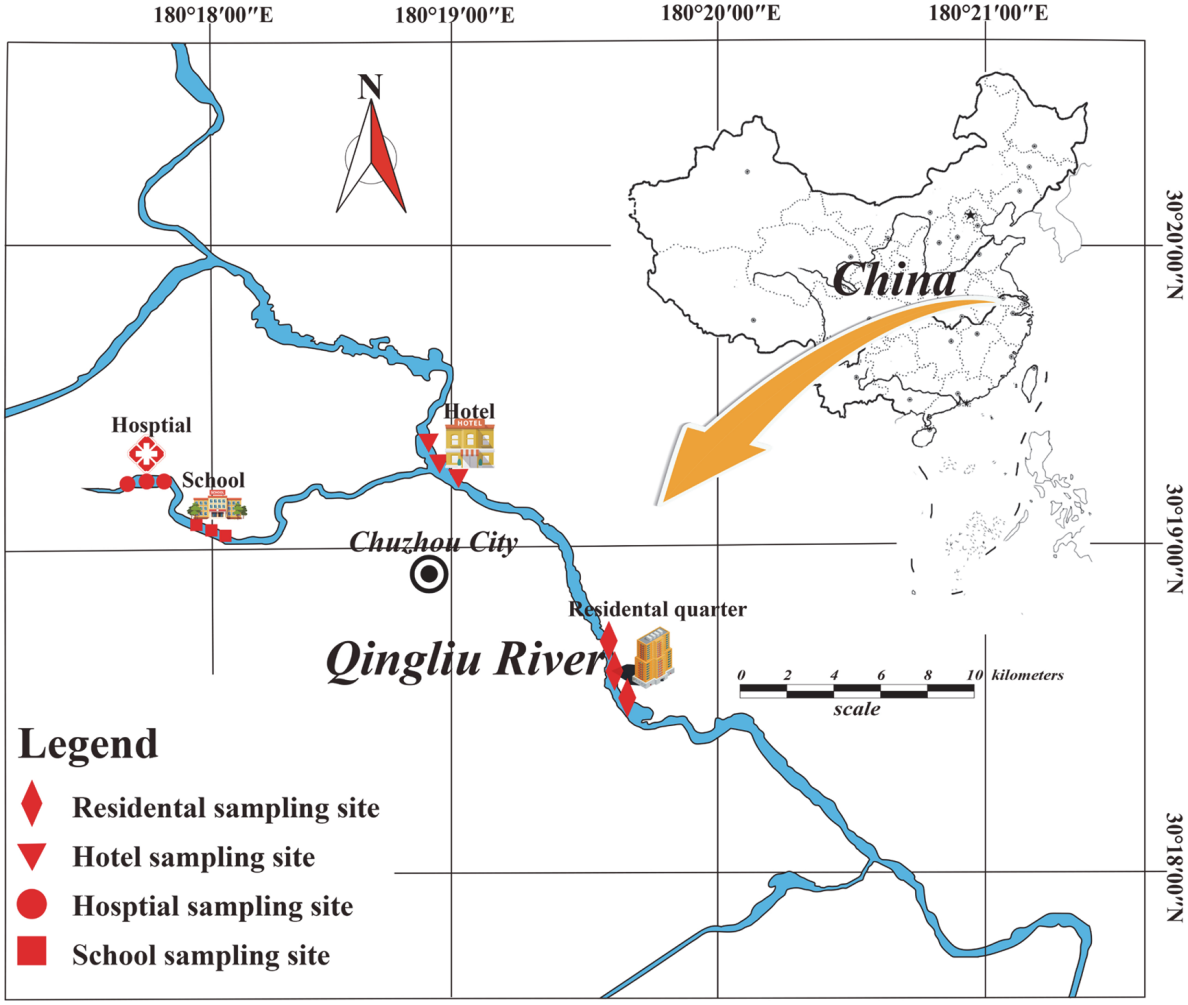
Map showing the location and distribution of sampling sites across the urban region of Qingliu River (Chuzhou section).

The sampling sites were located in the Qingliu River (watershed area of 1,265 km^2^ and mainstream length of 84.1 km), a typical urban river in Chuzhou City, China. This river is surrounded by residential and commercial areas, throughout the entire city of Chuzhou from the northwest to the southeast (Fig. 1), and it finally enters the Yangtze River through the Chu River. Excessive domestic sewage input results in the deterioration of this urban river. Sediment pollution under the long-term influence of domestic sewage has become an important issue in river ecosystem [37-39].

In this study, four representative areas affected by typical domestic pollution from the school (XX), residential quarter (JM), hospital (YY), and hotel (JD), were chosen as the sampling sites (Fig. 1). On the morning of May 6, 2019, three replicate sediment samples were collected at each sampling site, and these subsamples were obtained at 3 m intervals. The 12 sampling sites are shown in Fig. 1.The surface (< 5 cm in depth) sediment at each site was collected using a Peterson stainless steel grab sampler. Large fragments were removed, and the samples were stored in sterile polyethylene zipper bags, sealed, and transported to the laboratory on ice within 4 h. The samples were then homogenized completely, and subsamples were placed in 2.5 ml sterile centrifuge tubes. The remaining samples were kept at 4°C for immediate physicochemical analysis.

### Analysis of Physicochemical Properties

The temperature (T), dissolved oxygen (DO), and pH of the overlying water on the samples were measured with a multiparameter sonde (YSI 6600V2, USA) during sample collection. The total nitrogen (TN), total phosphorus (TP), and total organic carbon (TOC) contents were determined in the laboratory according to standard methods [38].

### DNA Extraction

A 0.25 g sediment sample was collected, and then genomic DNA from each sediment sample was extracted in triplicate using a DNA Isolation Kit (E.Z.N.A., Omega, USA) in accordance with the manufacturer’s instructions. Subsequently, the concentration and purity of genomic DNA were detected with an ultraviolet spectrophotometer, and verified by 1% agarose gel electrophoresis. The measured DNA sample was stored at -20°C for 16S rRNA gene PCR amplification and sequence analyses [40].

### PCR Amplification and Pyrosequencing

The V3-V4 region of the bacterial 16S rRNA gene was amplified using primers 338F (5′-ACTCCTACGGGA GGCAGCAG-3′) and 806R (5′-GGACTACHVGGGTWTCTAAT-3′). According to formal experimental conditions, all the samples were processed. The PCR products were mixed and examined by 2% agarose gel electrophoresis. The PCR products were purified using AMPure beads and finally entrusted to Majorbio Bio- pharm Technology Co., Ltd., Shanghai, China for sequencing.

### Sequencing Data Optimization and OTU Clustering

In this study, Illumina MiSeq sequencing was used to obtain double-end sequence data, which were optimized with fastp software. The details were as follows: First, pairwise reads were combined into a sequence following the overlapping relationships between PE reads, and the effects of the merge were filtered by quality control. Then, the samples were distinguished based on the barcode and primer sequences at both ends of the sequence. Obtaining an effective sequence and correcting the sequence direction were performed to optimize the data.

The optimized sequences were clustered into Operational Taxonomic Units (OTUs) using Usearch (version 7.1 http://drive5.com/uparse/) [41]. The RDP classifier (version 2.2 http://sourceforge.net/projects/rdp-classifier/) Bayesian algorithm was used to classify the 97% similarity level representative OTU sequences to obtain the species classification information corresponding to each OTU [42].

### Statistical Analysis

Based on the OTU information, the alpha diversity index of differences in community composition (Chao 1, ACE, Shannon, Simpson) and a sequencing depth index (Good's coverage) were calculated using Mothur software (version v.1.30.1 http://www.mothur.org/wiki/Schloss_SOP#Alpha_diversity) [43], and rarefaction curve graphs were generated with the R language tool (https://cran.R-project.org/web/packages/Vegan/) [44]. Beta diversity was represented by Bray-Curtis distance matrices generated from the OTUs table and statistical analysis and mapping using principal coordinate analysis (PCoA) in R language [45, 46]. According to the results of the taxonomic analysis, the community structure composition at different classification levels (such as the phylum, genus, and OTU, etc.) could be obtained. Based on the Kruskal-Wallis ranking and test, using the state package of R and the scipy package of Python to generate histograms, a significant difference test was performed between groups [47]. A similarity analysis (ANOSIM) was performed with the Bray-Curtis similarity matrix of the original pyrophosphate/phosphate data using the R-language vegan software package [48]. The relationship between the bacterial community structure and environmental variables was assessed using the Redundancy discriminant analysis (RDA) vegan package in R language [49]. The prediction of bacterial function and metabolic pathways was performed using Phylogenetic Investigation of Communities by Reconstruction of Unobserved States (PICRUSt) [35, 50]. Based on the correlation between phylogeny and functions, PICRUSt was used to obtain information at different pathway levels (levels 1–3) and a table of the abundance of each level [88]. The co–occurrence networks of the microbial communities were determined via Spearman correlation. Gephi was used for network visualization and modularization analysis [51]. The network topology characteristics determined by Gephi include modularity, average path length, and the topology properties of each note include the degree and betweenness centrality [52]. Specific steps of the analysis were based on an online analysis platform (https://www.i-sanger.com/). The original sequencing data have been submitted to the NCBI Sequence Read Archive and the project accession code is SRP241215.

## Results

### Physicochemical Properties of the Sediment Samples

The physicochemical parameters at the different sampling sites are presented in Table S1. Overall, the pH value of the overlying water ranged from neutral to alkaline conditions (pH = 7.46 to 8.72). The total phosphorus (TP) content of the river sediments did not significantly differ under the different types of domestic sewage pollution. However, there was a large difference in total organic carbon (TOC), ranging from 0.49 to 4.73 mg/g and the samples from the hotel site (JDS) has the lowest content. In addition, the total nitrogen (TN) content of JDS was significantly less than the other samples. Notably, the contents of total phosphorus (TP), total nitrogen (TN), and total organic carbon (TOC) were higher in the school site sample (XXS) than the other samples.

### Diversity and Composition of the Bacterial Communities

**Fig. 2:**
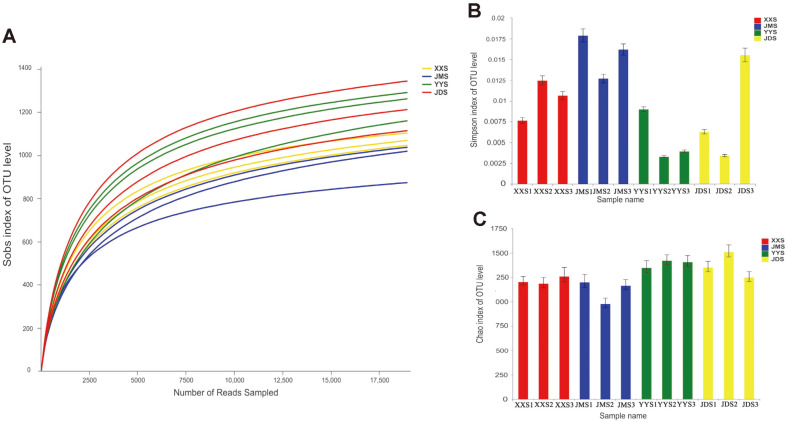
(A) Rarefaction curves of OTUs clustered at 97% sequence identity across the five samples. Properties of the bacterial communities of the twelve river sediments. (B) Diversity, expressed as the Shannon Index: Higher values indicate higher diversity. (C) Richness, indicated by Chao's richness estimator Chao 1: higher values indicate higher diversity.

In our study, the diversity and phylogenetic structure of the bacterial communities in the sediment samples were analyzed using Illumina MiSeq high-throughput sequencing. A total of 452,025 effective 16S rRNA gene sequences were acquired after removing low-quality sequences and chimeras. Of these, after the clustering and alignment of 12 sediment sludge samples, 1619 OTUs were obtained according to the standard of 0.97 similarities. The average coverage of all samples was 99.01 ± 0.1% (mean ± s.d.) (Table S2), indicating that the sequencing depth was sufficient for community analysis. All rarefaction curves verged toward saturation (Fig. 2A), indicating a high degree of confidence in the bacterial community structure. As seen in Table S2, the total number of OTUs in the four samples differed. The average total numbers of OTUs in the hospital samples (YYS) and hotel samples (JDS) were 1094 and 1056, respectively. The samples from the school site (XXS) and residential quarter site (JMS) had 920 and 838 OTUs, respectively. The results showed that YYS had the highest species richness and that JMS had the lowest species richness. The change of Chao1 and ACE index was consistent with the total number of OTUs (Fig. 2C and Table S2), which confirmed the foregone conclusion.

The Shannon and Simpson indices were used to estimate the microbial diversity in the samples [1]. The Shannon index values for the YY and JD samples were higher than those for the XXS and JMS samples (YYS and JDS were 6.71 and 6.67, respectively, and XXS and JMS were 6.30 and 5.9, respectively) (Table S2). The Simpson index for JMS was the largest (0.0123), and that for YYS was the smallest (0.0039); the values for XXS and JDS were 0.0078 and 0.0061, respectively (Table S2). Consequently, the YYS and JDS had a high, and XXS and JMS, a low species community diversity.

**Fig. 3:**
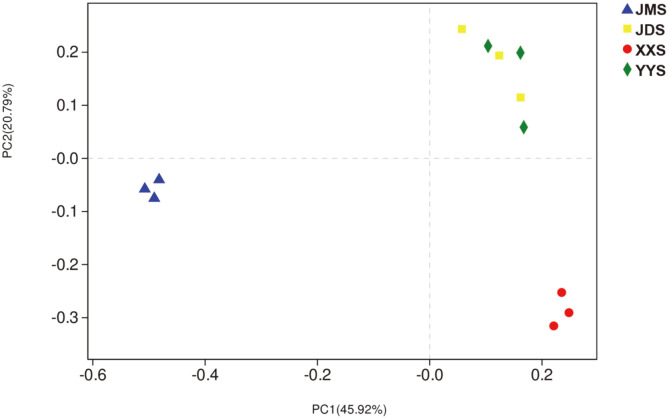
PCoA analysis of sedimentary bacterial community diversity.

The PCoA was used to observe the composition of the various bacterial communities (Fig. 3). PC1 and PC2 accounted for 20.79% and 45.92% of the variation, respectively. The XX, YY, and JD samples were gathered on the right side of the figure, and JMS was distributed in the middle. Generally, the closer two samples were plotted, the more similar was their composition. It is worth noting that JMS was far from other samples in the graph, indicating a large difference in bacterial community composition from the other samples. In addition, ANOSIM indicated that the bacterial community structure of river sediment significantly differed among the different types of domestic sewage pollution (ANOSIM R = 1, *p* = 0.001).

**Fig. 4:**
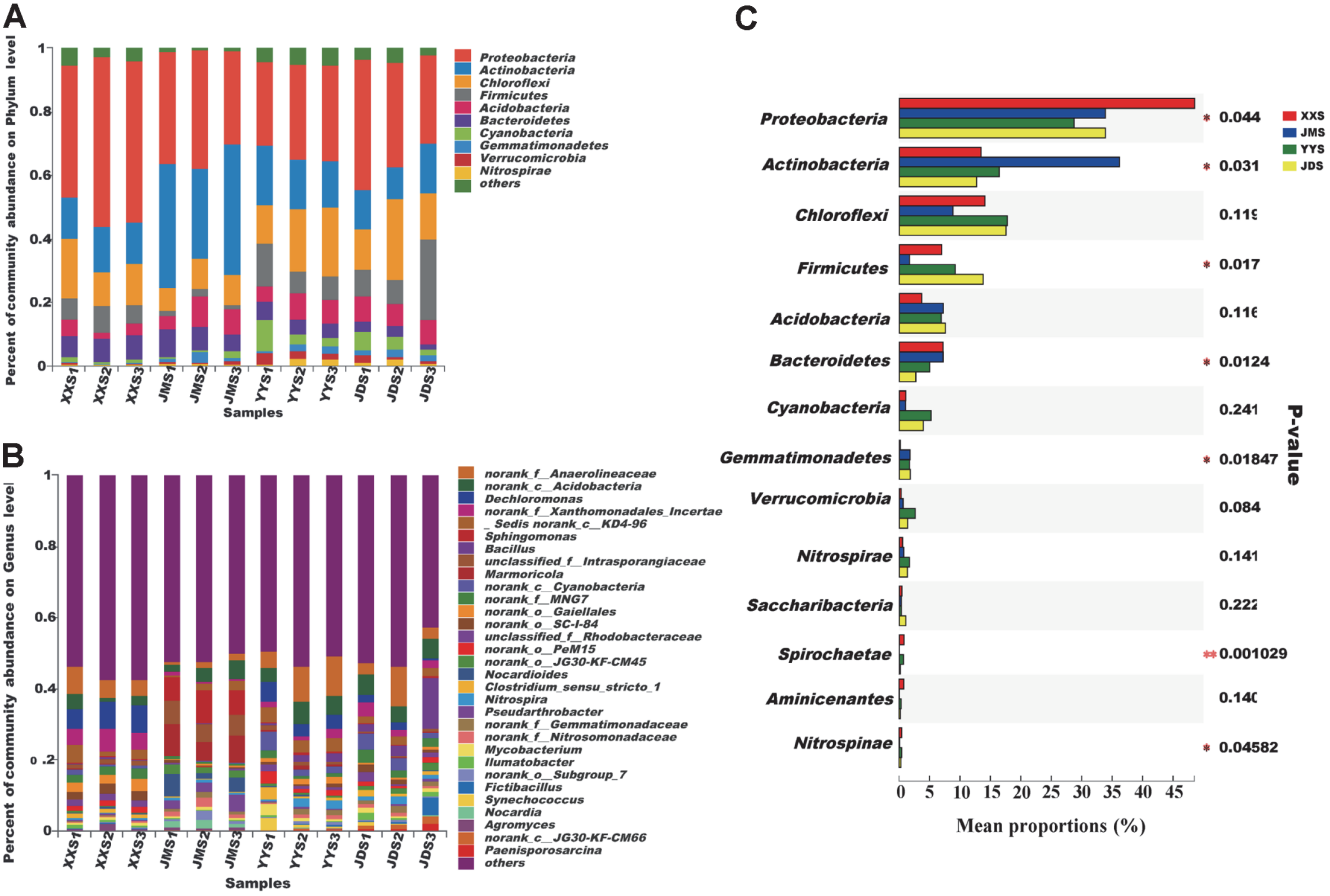
Community composition of bacteria in the phylum level (A) and the genus level (B) in sediment samples. (C) Comparison of bacterial abundances in river sediments under the influence of domestic sewage at the phylum level.

The sequences of the samples were sorted at the phylum classification level, as shown in Fig. 4A. Thirteen different phyla and unclassified bacterial members were detected in the urban river sediment samples. Proteobacteria, Actinobacteria, Chloroflexi, Firmicutes, Acidobacteria, Bacteroidetes and Cyanobacteria (relative abundance > 1% at least in a sample) were the dominant bacteria in all samples, accounting for 83.98% to 93.89% of the total sequences. Proteobacteria was the most abundant phylum in the XXS, YYS and JMS, with relative proportions of 48.5%, 28.74%, and 33.89%, respectively. This dominant bacterial taxon was the second highest in JMS, accounting for 28.74% of the total. Actinobacteria had the highest content in JMS, accounting for 36.18% of the total community. In addition, compared with the other samples, Firmicutes had the lowest content in JMS, accounting for 1.67% of the total community. It is worth noting that Proteobacteria, Actinobacteria, Firmicutes, Bacteroidetes, Gemmatimonadetes, and Nitrospirae were identified in the four sample types (*p* < 0.05) (Fig. 4C).

**Fig. 5:**
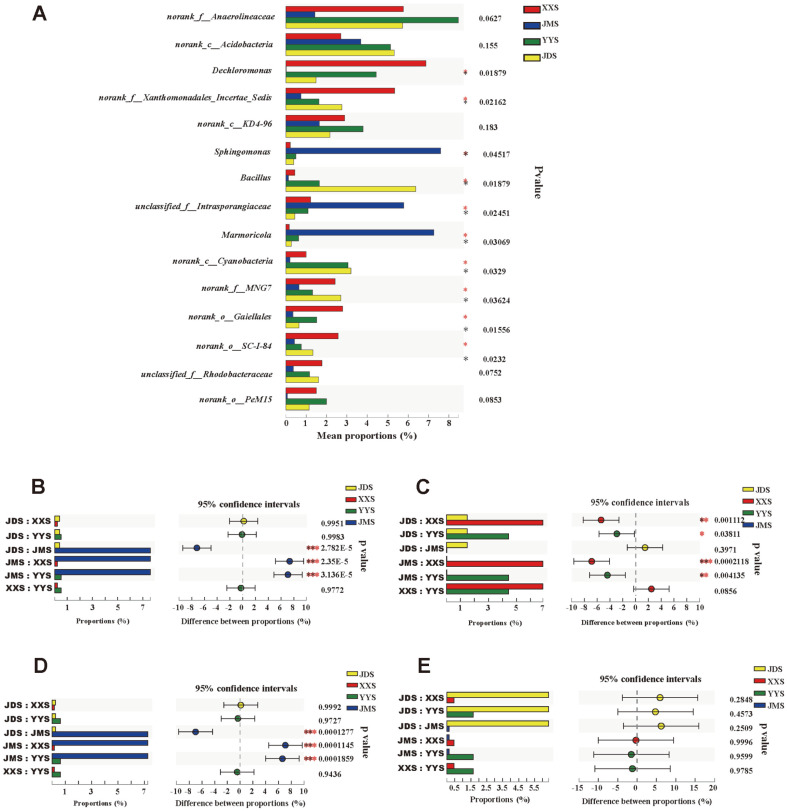
Comparison of bacterial abundances in river sediments under the influence of domestic sewage at the genus level (A). One-way ANOVA bar plot on genus level for *Sphingomonas* (B), *Dechloromonas* (C) and *Marmoricola* (D), and *Bacillus* (E) * stands for 0.01 b *p* ≤ 0.05, ** stands for 0.001 b *p* ≤ 0.01 and *** stands for *p* ≤ 0.001.

To further reveal the structure of the bacterial communities in the river sediments, the relative abundance and classification of OTUs were analyzed at the family and genus levels (Figs. S1 and 4B). When comparing the species composition of a particular bacterial family at all sampling locations at the taxonomic level, the difference in JMS was more pronounced. *Nocardioidaceae, Sphingomonadaceae, Intrasporangiaceae,* and *Xanthomonadaceae* were the dominant families (Fig. S1). At the genus level, *Dechloromonas, Nitrospira, Mycobacterium, Ilumatobacter, Bacillus* and *Pseudarthrobacter* were the richest bacterial taxa in the sediment samples (Fig. 4B**)**. These bacteria were also the common genera in XXS, YYS, JMS, and JDS. Notably, *Nocardia* (3.83%)*, Pseudarthrobacter* (2.97%), *Marmoricola* (7.26%), and *Nocardioides* (4.05%) were unique genera in JMS. *Dechloromonas* was a unique species in XXS, with a relative proportion of 6.87%. In addition, *Sphingomonas* was the main genus in JMS, with a relative abundance of 7.59%. The genus *Agromyces* was found in XXS (0.88%), YYS (0.32%), JMS (0.48%), and JDS (0.28%). We observed that 10 of the 15 genera showed statistically significant differences among the four groups (Fig. 5A). We found statistically significant differences in *bacillus* among the four groups, but not between the two groups (Fig. 5E). *Marmoricola* (Fig. 5D), *Dechloromonas* (Fig. 5C), and *Sphingomonas* (Fig. 5B) showed statistically significant differences among the four sample types and the two groups.

### Co–Occurrence Networks

**Fig. 6:**
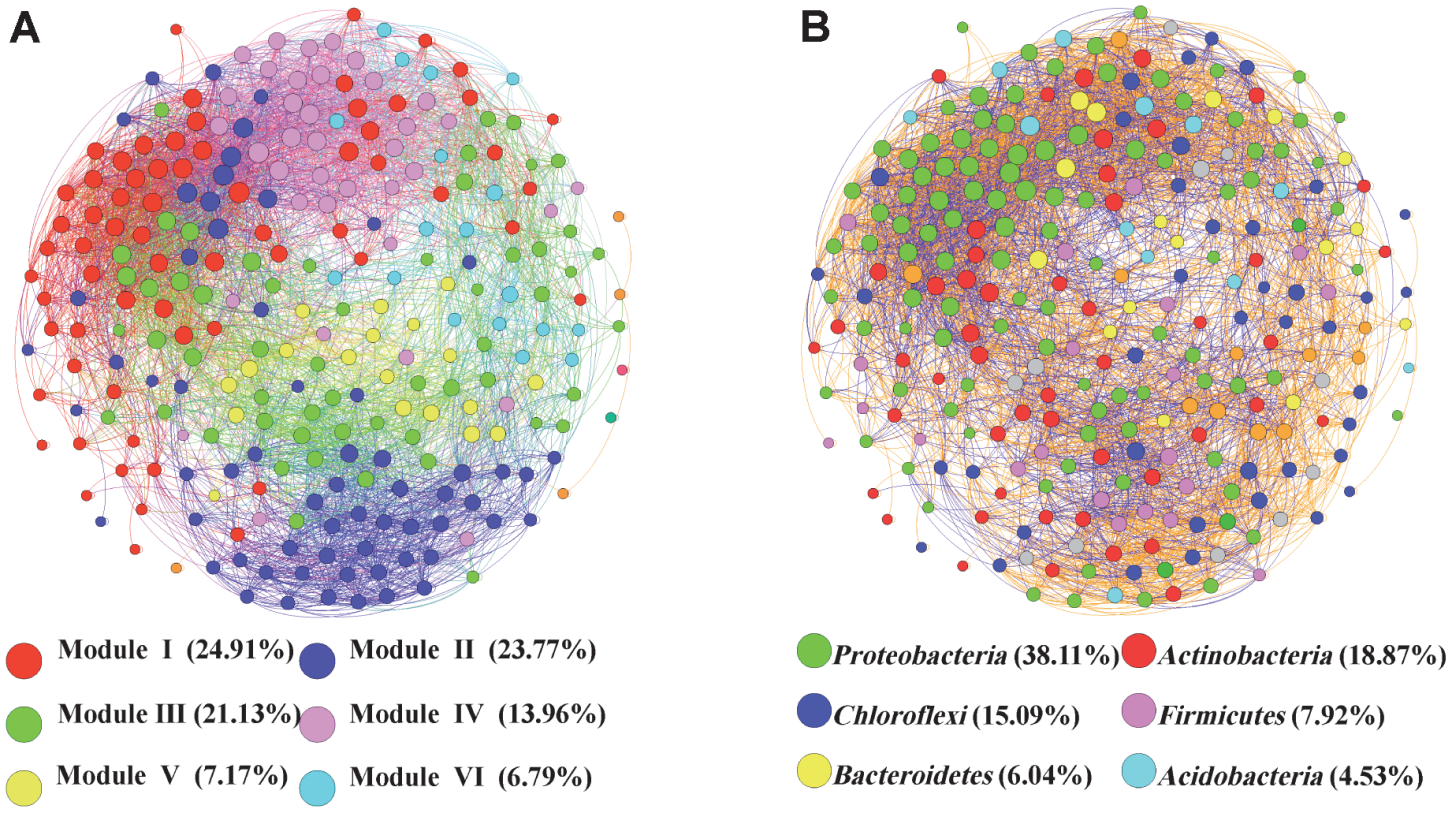
Spearman’s correlation-based network analysis between OTUs for the inflow rivers samples. The size of each node is proportional to the number of connections (*i.e*., degree), and the nodes are colored according to different types of modularity classes (**A**) and phylum (**B**), respectively. Colored nodes (except light gray) represent the six major groups and light gray represents all modules except the six main major modules. Orange and blue edges indicate positive and negative correlations.

Considering the nonrandom community aggregation model of bacteria under different levels of domestic sewage pollution, a network interface was built to exhibit the topological and taxonomic characteristics of the bacterial co–occurrence patterns in river sediment (Fig. 6). In accordance with the Spearman correlation analysis, the sediment bacterial network in the river was composed of 265 nodes (*i.e*., OTUs) and 446 edges, and there was a strong correlation between nodes and nodes in pairs (Spearman’s ρ ≥ 0.7, *p* < 0.05) (Fig. 6). In addition, the analysis showed that the topological characteristics of the bacterial communities in river sediments could be distinguished among different levels of domestic pollution. The networks exhibited higher values of modularity (MD, 0.635; Values> 0.4 indicate that the network is modular) and the Clustering coefficient (CC) than the global network of all sampled sites, suggesting that the different pollution sites had obvious co–occurrence patterns and modular structures.

Modularity analysis showed that the whole network could be divided into six main modules (Fig. 6A). In these modules, species interactions were more frequent and intense than in the rest of the community. The OTUs from modules II and VI had the highest relative abundances in YYS, while those from modules I and IV tended to occur in XXS. The OTUs of modules II and VI were relatively high in abundance in YYS, while those from modules I and IV tended to be high in abundance in XXS. However, the classification relationship was obviously a critical factor in determining the module structure in the network. The nodes in modules I and IV were mostly Proteobacteria. The nodes in module II belonged to Proteobacteria, Firmicutes and Chloroflexi. The nodes in module III mostly belonged to Actinobacteria and Proteobacteria. The nodes in module V were mainly Cyanobacteria. Unclassified *Anaerolineaceae* dominated module VI (16.67%), while unclassified Cyanobacteria dominated module V (15.79%). According to the betweenness centrality scores, *Variibacter, Parasegetibacter,* unclassified *Caldilineaceae*, unclassified *Nitrosomonadaceae* and unclassified *Geodermatophilaceae* were identified as the top five genera. This finding suggested that these bacteria played a key role as keystone taxa within the co-occurrence network.

### Environmental Factor Analysis

**Fig. 7:**
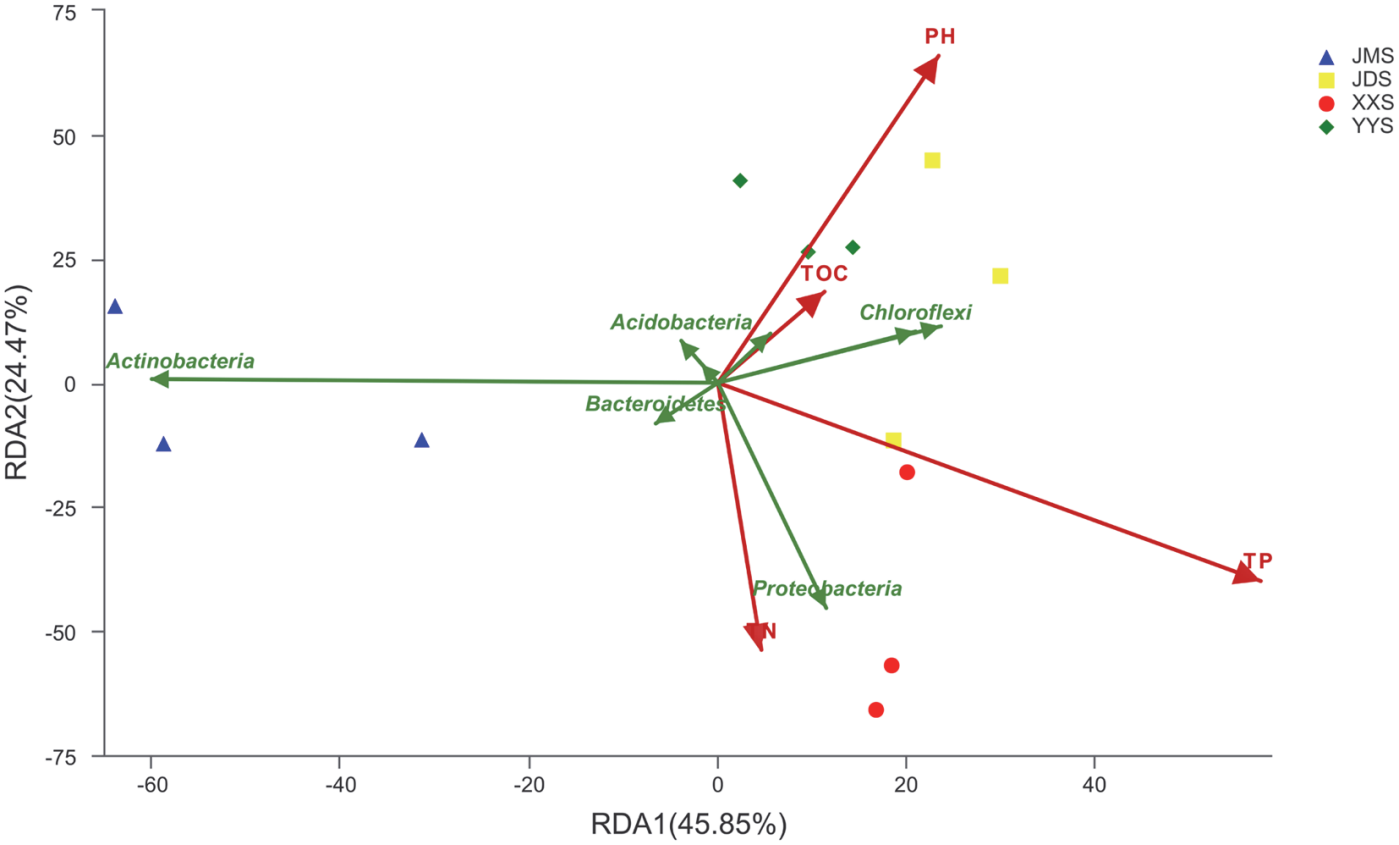
RDA analysis of bacterial community species (phylum) in sediments and environmental factors.

Environmental factors are an important driver of the structure of the microbial community [53]. RDA was performed at the phylum level to identify the relationships between the composition of the bacterial community and environmental parameters, as shown in Fig. 7. RDA1 and RDA2 accounted for 74.91% and 21.74% of the interpretations, respectively. Notably, TP (*p* = 0.001) and pH (*p* < 0.005) were the environmental variables affecting the environmental relationships of bacterial communities, and TN (*p* > 0.05) and TOC (*p* > 0.05) had weaker effects. Furthermore, Actinobacteria, Bacteroidetes and Acidobacteria were positively correlated to pH, TN, and TOC and negatively correlated with TP. Interestingly, Proteobacteria and Cyanobacteria were positively related to all environmental factors. The most abundant phylum, Actinobacteria, was negatively related to pH, TOC, and TP but had little relationship with TN.

### PICRUSt Functional Predictive Analysis

**Fig. 8:**
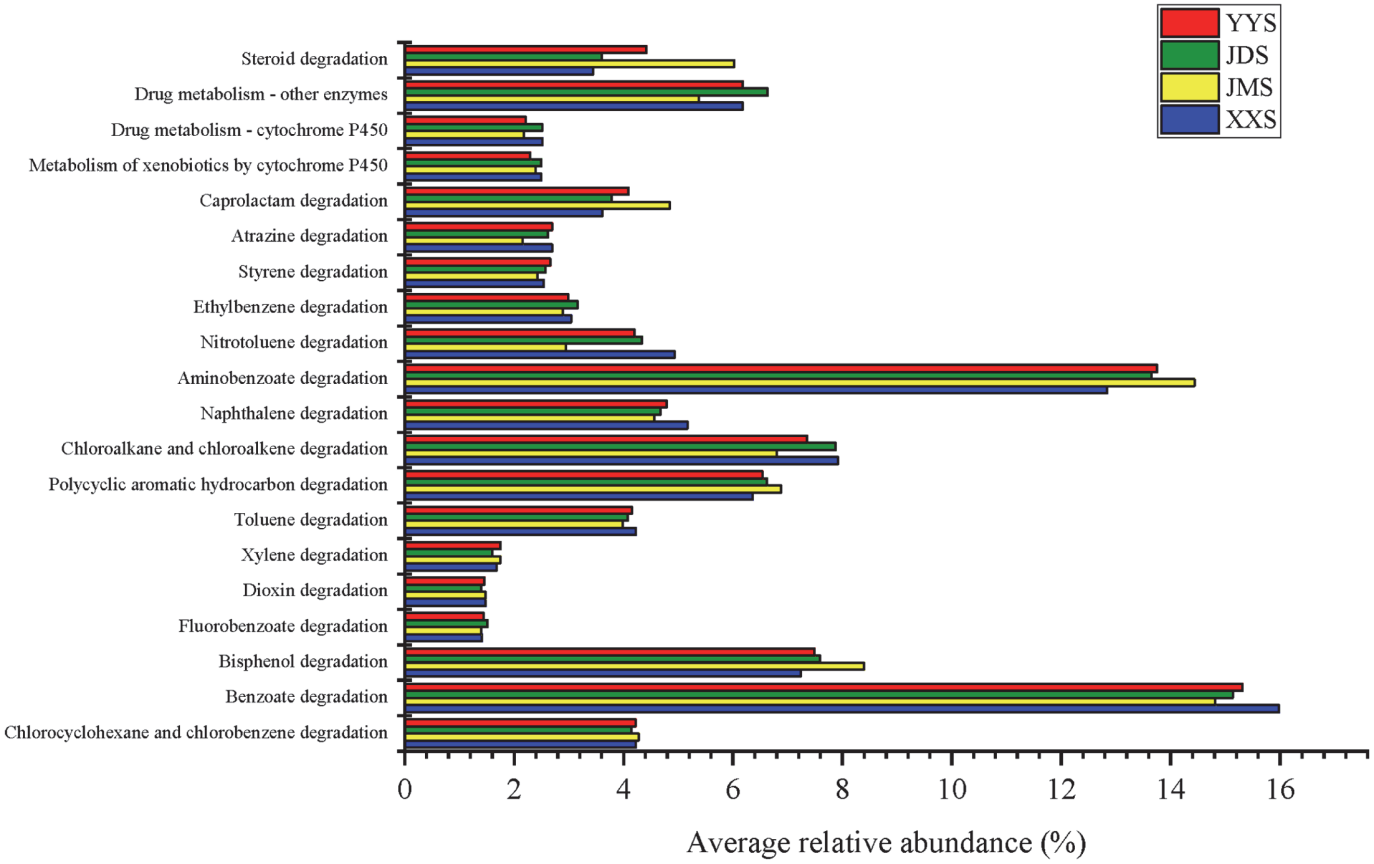
The comparison of bacterial community functions predicted by PICRUSt at level 3 related to the degradation and metabolism of xenobiotics between different groups with the influence of domestic sewage.

To understand the function of river sediment bacteria under domestic sewage pollution, this study used PICRUSt software for prediction by comparing the relative abundance of the four groups in functional categories (level 1, Fig. S2), subcategories (level 2, Fig. S3), and individual pathways (level 3, Fig. 8). The results showed that the major functional gene families of all samples were involved in Metabolism, Environmental Information Processing, Genetic Information Processing, Cellular Processes, Human Diseases and Organismal Systems. It is worth noting that there was a high abundance of genes related to metabolism in bacterial communities of XXS, YYS, JMS and JDS (Fig. S2). Therefore, to further determine the functional differences, we compared the relative abundance of genes related to metabolism at level -2. As shown in Fig. S3, the genes involved in amino acid and carbohydrate metabolism were present in all samples with the highest abundance. In addition, the samples from residential quarter (JMS) had a relatively high abundance of genes related to xenobiotics biodegradation and metabolism, lipid metabolism and terpenoid and polyketide metabolism (Fig. S3). In general, rivers are the main recipients of pollutants and xenobiotics, wherein most aquatic organisms and bacteria are exposed to these xenobiotics [1, 54]. Among the metabolic genes and pathways associated with xenobiotics biodegradation at level-3, the genes related to benzoate and aminobenzoate biodegradation were the most abundant genes in JMS and XXS (Fig. 8). Samples with an abundance of predictive genes related to Bisphenol degradation were found in JM (Fig. 8).

## Discussion

Freshwater ecosystems are particularly vulnerable to the surrounding environment [1, 27, 55]. Fluctuations caused by various pollutants accelerate the energy flow and material circulation in the river ecosystem, which may have a direct impact on the microbial community in the river. Our research showed significant differences in the bacterial diversity of river sediments under different types of domestic sewage pollution (Table S2). A relatively low bacterial diversity was detected in samples XXS and JMS, potentially due to the massive enrichment of some species, which are very suitable for conditions of sewage pollution in schools and residential areas. In addition, JMS showed the lowest species diversity (Table S2). The main reason may be related substances (such as household chemicals, fungicide and pharmaceutical residues) in the complex living environment of the residential area, which inhibits the activity of certain bacteria in the river [87, 88]. PCoA (Principal co-ordinates analysis) showed that the bacterial community structure of YYS (the samples from the hospital) and JDS (the samples from the hotel) had more similarities (Figs. 3 and 4A). This phenomenon may mean that many taxa are generalists, able to utilize multiple nutrients [17], and/or able to tolerate severe environmental disturbances [59]. Based on the above results, the bacterial community is driven by various environmental factors, which indicates that the bacterial communities in river sediments may have developed phylogenetic diversity to cope with pollutants from different sources.

The bacterial communities in our study included aquatic microorganisms and microorganisms of human origin, and the bacterial compounds from different presumptive sources also differed among sampling locations. By analyzing sediments under the influence of domestic sewage in this study, Proteobacteria, Actinobacteria, Chloroflexi, Firmicutes, Bacteroidetes, and Acidobacteria were found to be the most abundant phyla (percent of sequences). Consistent with other studies, these members are usually detected in freshwater sediment environments [60-62]. Although most bacterial groups are present in sediments, a comprehensive comparison revealed differences among the dominant bacteria in the samples (Fig. 4A). Historically, Actinobacteria were considered to be the primary active resident taxon in soils [63]. However, according to the data in this study, the main phylum of gram-negative bacteria, Proteobacteria, was the most abundant phylum involved in various biogeochemical processes in aquatic ecosystems (*e.g*., carbon and nitrogen cycles) [38]. In addition, RDA showed that the Proteobacteria in the river sediment was directly proportional to TN and TP (Fig. 7), which may have been caused by the high nutrient content in the sewage. This observation indicated that a high abundance of Proteobacteria is related to high nutrient utilization [56, 57]. Among all the classification results, the main sedimentary bacterial populations in the samples from the residential quarter (JMS) were the most significantly different (Fig. 4A), mainly including taxa related to nutrient contamination and other anthropogenic disturbances. The families *Nocardioidaceae*, *Sphingomonadaceae, Chitinophagaceae* and *Intrasporangiaceae* were dominant at the JM site. *Sphingomonadaceae* are often found in habitats polluted by recalcitrant aromatic compounds from natural or anthropogenic sources [64] (Fig. S1). Specifically, these populations have the ability to degrade recalcitrant high-molecular-weight compounds [65]. Members of *Chitinophagaceae* can degrade chitin and cellulose [66]. *Nocardioides* species are chemoorganotrophs and utilize a variety of carbon and nitrogen sources [67]. Therefore, we conclude that the relative advantages of these high-abundance organisms reflect that the carbon cycle plays an important role at the JM site.

In addition, nutrient conditions may select certain bacterial species, resulting in differences in the distribution of bacterial populations. The genus *Dechloromonas* was the most dominant group at the XX and YY sites. This genus is commonly found in freshwater sediment environments and can oxidize aromatic compounds such as toluene and chlorobenzoate [48]. Hospitals represent an irrefutable source of the release of numerous compounds into the aquatic environment due to laboratory activities or the release of pharmaceuticals into wastewater [70]. Therefore, the relative dominance of the genus *Dechloromonas* at the XX and YY sites may reflect the presence of high levels of organics. Acidobacteria and Bacteroidetes also showed good properties in the samples from the school and hotel (XXS and YYS, respectively) (Fig. 4A). Acidobacteria are renowned for their resistance to contaminants such as petroleum compounds [71, 72]. *Bacteroides* are gram-negative heterotrophic bacteria that are commonly found in freshwater ecosystems. They are ubiquitous in organic-rich systems and are considered to be adept at the degradation of high-molecular-weight organics in different ecological environments [73-75]. The RDA showed that Acidobacteria were positively correlated with YYS, Bacteroidetes were positively correlated with XXS, and the XXS and YYS samples were rich in TOC (Table S2). Therefore, the presence of Acidobacteria and Bacteroidetes further confirmed the prevalence of organic compound waste in areas where YY and XX deposits were located. These observations are consistent with other studies, showing changes in bacterial communities caused by different types of wastewater discharge [17, 38].

Network analysis can help explain the basic network of microbe-microbe interactions in addition to the changing bacterial community diversity and composition. The analysis of habitat networks in this study provides a visualization of the mutuality of bacterial genera and valuable supplementary information for the study of river sediment communities under the influence of domestic sewage (Fig. 6A). According to the betweenness centrality scores, the genera *Variibacter, Parasegetibacter,* unclassified *Caldilineaceae,* unclassified *Nitrosomonadaceae* and unclassified *Geodermatophilaceae* were identified as the top five, indicating that they may play a key role in maintaining the structure and function of sediment ecological communities in rivers. The genus *Variibacter* is a strictly aerobic bacterial taxon that provides essential information for understanding bacterial activities associated with the growth of plants [76]. The genera *Parasegetibacter* and unclassified *Caldilineaceae* have potential nitrification and phosphorus accumulation abilities [39, 77]. Unclassified *Nitrosomonadaceae* are closely related to N cycling. Unclassified *Geodermatophilaceae* members have properties that inhibit the production of reactive oxygen species and play a key role in extreme environments where nutrient supplies are scarce [78].

By integrating the classification allocation of OTU nodes and network structure, we found that closely related categories tended to interconnect and gather together (Fig. 6B), which can be explained by the overlapping strong niches in closely associated species or synergistic relations. In our study, the nodes were divided into four main modules, and different modules responded to different functions [79]. A strong ecological connection of co- occurrence cluster correlations was observed. In module IV, certain bacteria were related to the degradation of organic carbonate. For example, unclassified *Anaerolineaceae*, which are dominant in YYS, play a key role in the initial activation of long-chain alkane biodegradation [80], and can anaerobically decompose carbohydrates via fermentation [81]. Therefore, the large presence of unclassified *Anaerolineaceae* in module IV indicated that the organic matter in YY controlled these bacterial activities. In module II, the genus *Bacillus* was relatively abundant in JDS (Fig. 4B). Urban sewage, especially domestic sewage, has been confirmed to contain a large number of pathogenic microorganisms, including pathogenic bacteria and enteroviruses [82, 83]. In this study, the genus *Bacillus* was abundant in JDS. These bacteria are potentially pathogenic and can be found in fresh and aging feces [84]. There is no doubt that the organic and even toxic substances in JD wastewater have a significant impact on the role of the genus *Bacillus* in the modular structure. Conversely, the major taxa in module II likely participate in biogeochemical N cycles. *Nitrospira* species are the dominant nitrite oxidizers within freshwater sediments [85]. The genus *Paenisporosarcina* is associated with the function of nitrate reduction [10], and *Comamonadaceae* are known denitrifiers [86]. Therefore, the complexity of community structure, as well as the co-occurrence patterns of bacterial communities in river ecosystems affected by domestic sewage, seems to be nonrandom and functionally driven.

Based on PICRUSt prediction, this study revealed the potential functional characteristics of bacterial communities in river sediments caused by domestic pollution. A relationship between xenobiotic degradation genes and xenobiotic biodegradation rates has been suggested. Additionally, the biodegradable genes could be used as indicators for the existence of xenobiotics and their metabolites [89, 90]. In this study, genes related to benzoate and aminobenzoate biodegradation were the most abundant xenobiotic degradation genes in all the samples. As the key mediator in PAH biodegradation, there are a large number of genes related to benzoate and aminobenzoate biodegradation, indicating that the areas polluted by domestic sewage contain persistent organic pollutants with high contents from both JMS and XXS. This functional response of the bacterial community may accelerate bioremediation in polluted areas. Moreover, genes related to bisphenol degradation were detected in all river sediment samples (Fig. 8), and especially those from JMS with the highest value (8.19%), indicating that organic toxic chemicals may be contained in untreated wastewater from JMS. Bisphenol has been shown to exert acute toxicity toward aquatic organisms and to disrupt their endocrine function [91, 92]. Therefore, direct discharge of domestic sewage into rivers may pose a high risk to the bacterial ecology.

In conclusion, this study revealed the structure, diversity, co-occurrence patterns, and metabolic functions of bacteria in the middle layer of urban rivers under the influence of different types of domestic sewage. Research has shown that different types of sewage in cities affect the bacterial community composition in rivers, and the difference in bacterial community composition is particularly significant in JMS areas. Moreover, we also found evidence of the same high abundance of bacterial communities in different locations (such as the high abundance of the genus *Bacillus* in JD and YY), as nutrient factors may affect the bacterial community assemblage within the river sediment. The occurrence of nonrandom co-occurrence and ecological function-driven modular patterns provides a new perspective of microbial assembly in terms of the effects of pollution on the sediment bacterial community. Our results indicate that TP is a more important factor affecting the distribution of bacteria in river sediments under the influence of domestic sewage. This study provides insights into our understanding and monitoring of pollution in the aquatic environment and contributes to the management of river environments.

## Supplemental Materials



Supplementary data for this paper are available on-line only at http://jmb.or.kr.
