# Supplementary material for: Sediment Bacterial Community Structure under the Influence of Different Domestic Sewage Types
Source: J Microbiol Biotechnol. 2020 Jun 24;30(9):1355–66. doi: 10.4014/jmb.2004.04023 (PMC9728189; doi:10.4014/jmb.2004.04023)
Supplement: Supplementary file 1 [file JMB-30-9-1355-supple.pdf]

J. Microbiol. Biotechnol.
https://doi.org/10.4014/jmb.2004.04023

Supplementary materials

Table S1. Physicochemical parameters of sediments and overlying water within Qingliu River

Physicochemical parameters							
Surface water				Sediment			
**Samples[b]**	**T (°C)**	**pH**	**Do (mg/L)**	**Samples[b]**	**TN (mg/g, dry mass)a**	**TP (mg/g, dry mass)a**	**TOC (mg/g, dry mass)a**
**XXW**	27.0	7.47	8.73	**XXS**	1.62±0.05	0.84±0.04	4.77±0.05
**JDW**	26.5	8.26	8.15	**JDS**	0.57±0.07	0.51±0.02	0.53±0.04
**YYW**	27.4	8.72	9.56	**YYS**	1.17±0.04	0.59±0.05	1.93±0.08
**JMW**	26.0	7.67	7.49	**JMS**	1.02±0.06	0.35±0.05	0.96±0.08

Note: T, Temperature; DO, Dissolved Oxygen; TN, Total Nitrogen; TP, Total Phosphorus; TOC, Total Organic Carbon.

[a] Values are given as mean ± standard deviation (n = 3).

[b] The XXW, JDW, YYW and JMW represent water samples collected from the sampling site of school, hotel, hospital and resident quarter. The XXS, JDS, YYS and JMS represented sediment samples which were sampled in the same sampling site XXW, JDW, YYW and JMW.

Table S2. Estimates of richness and diversity for operational taxonomic units (OTUs) definition of 97% similarity for twelve river sediment samples obtained from urban rivers polluted by domestic sewage

Sample	OTUs	Shannon	Simpson	ACE	Chao 1	Coverage
**JMS1**	884	5.4044	0.0179	1166.03	1201.88	0.9889
**JMS2**	747	5.5492	0.0127	947.92	977.81	0.9931
**JMS3**	884	5.4949	0.0162	1152.34	1166.69	0.9904
**XXS1**	946	5.9295	0.0076	1182.17	1205.03	0.9920
**XXS2**	904	5.6221	0.0125	1165.87	1185.51	0.9901
**XXS3**	911	5.7353	0.0107	1180.62	1262.16	0.9901
**YYS1**	1030	5.6772	0.0089	1324.58	1347.63	0.9871
**YYS2**	1136	6.3423	0.0033	1391.48	1422.75	0.9901
**YYS3**	1117	6.2555	0.0039	1369.48	1408.26	0.9897
**JDS1**	1055	6.0116	0.0063	1326.92	1351.72	0.9895
**JDS2**	1149	6.4058	0.0034	1449.13	1510.12	0.9894
**JDS3**	965	5.6628	0.0155	1230.48	1249.41	0.9899

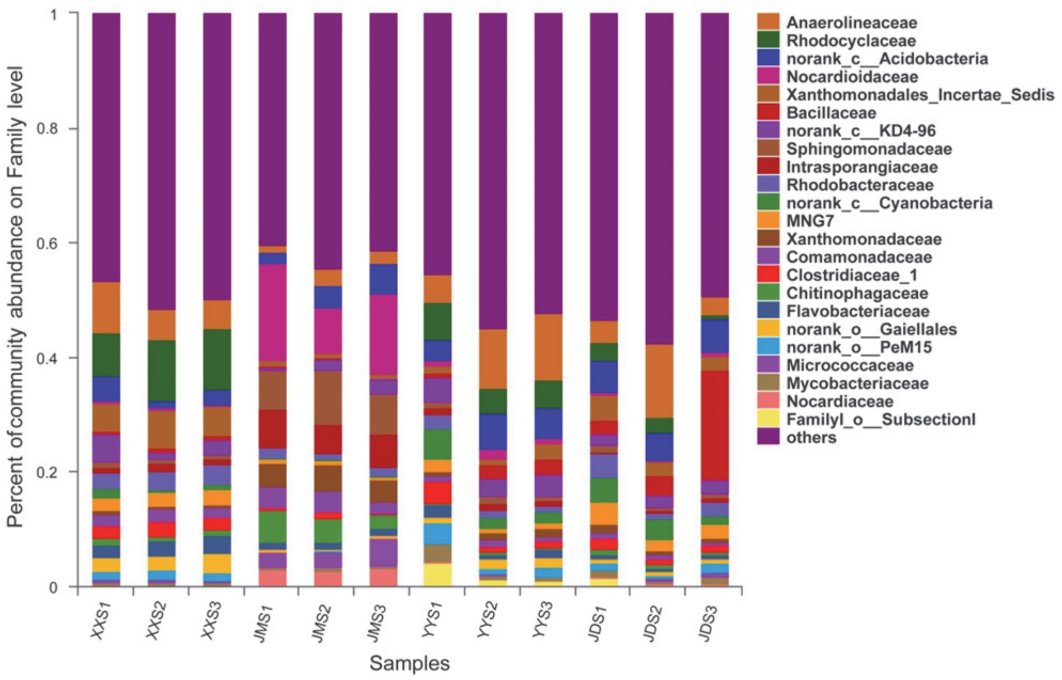

Figure. S1. Community composition of bacteria in the family level

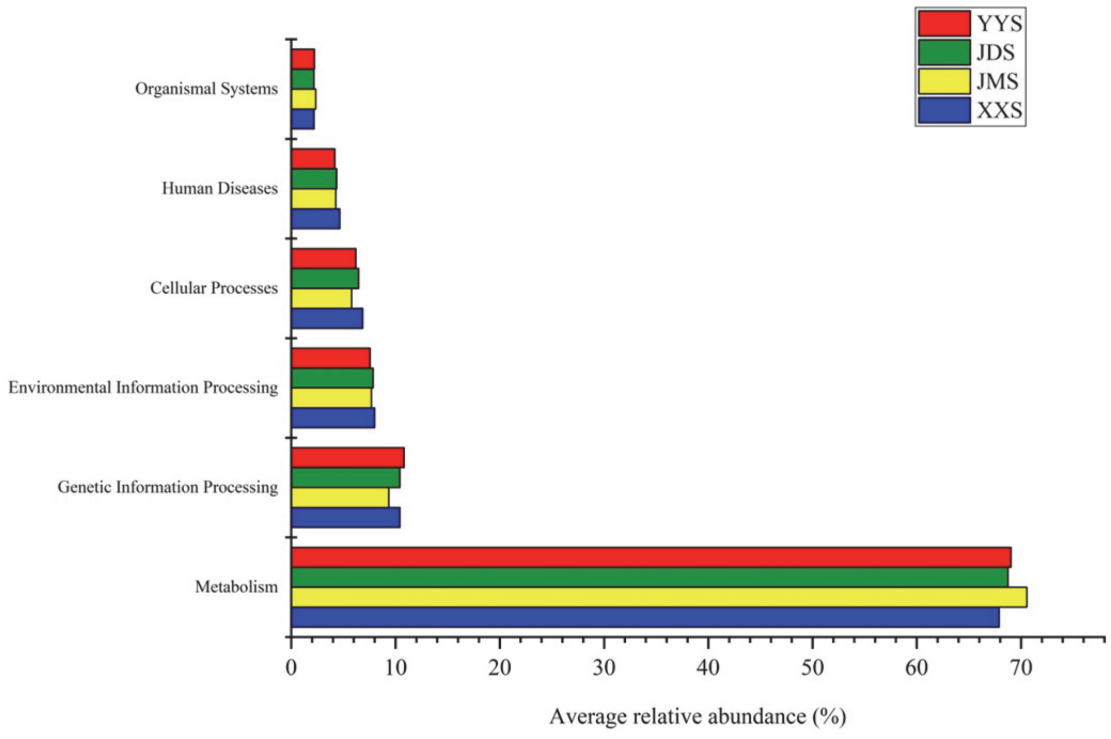

Fig. S2. The comparison of bacterial community functions predicted by PICRUSt at level 1 between different groups with the influence of domestic sewage.

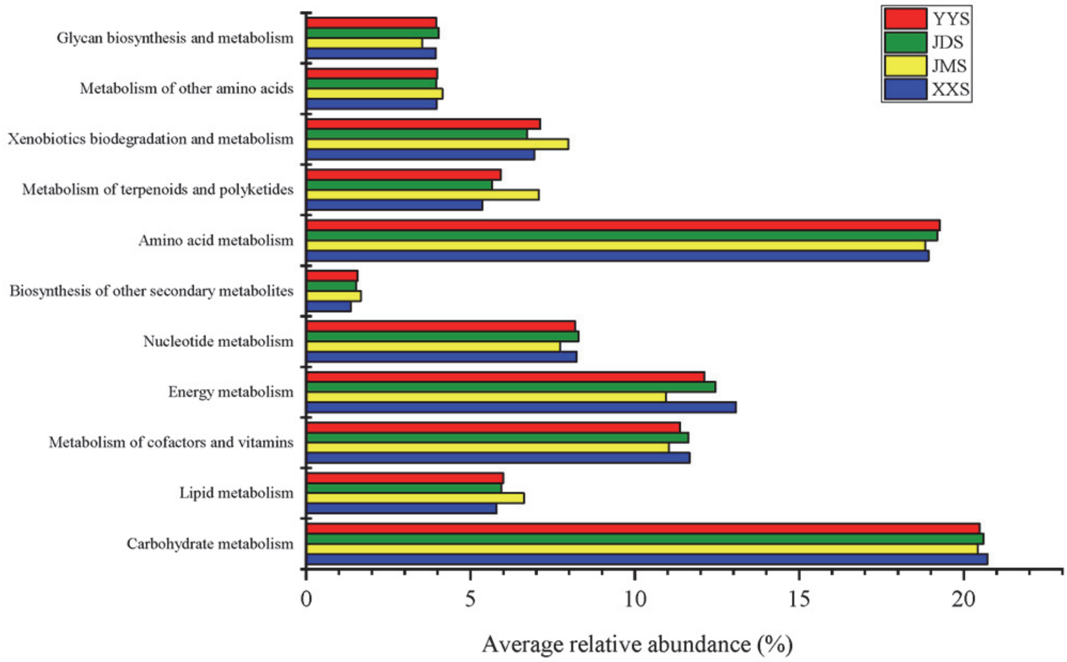

Fig. S3. The comparison of bacterial community functions predicted by PICRUSt at level 2 between different groups with the influence of domestic sewage.
